# Voluntary male mentors' lived experience of social engagement with men in their community

**DOI:** 10.1111/hsc.14067

**Published:** 2022-10-20

**Authors:** Mark Henderson, Mark Hughes, John Hurley, Gregory Smith

**Affiliations:** ^1^ Faculty of Health Southern Cross University Coffs Harbour Australia; ^2^ Faculty of Business Law and Arts, Southern Cross University Coffs Harbour Australia

**Keywords:** authentic, community, connection, empathy, male, mentor, volunteer

## Abstract

Men volunteering to mentor other men is a growing form of social engagement in Australia. Masculine norms associated with not disclosing emotional distress or discussing loneliness are often set aside by participating in these one‐to‐one relationships. Mentors have reported improvement in their well‐being and a desire to contribute more. In this phenomenological study, which draws on hermeneutic methodology, 12 men who voluntarily met and mentored another adult man for a minimum of 6 months participated in a semi‐structured interview. Findings showed non‐judgement and deep listening facilitated a rewarding and personally developmental relationship. Most voluntary mentors experienced substantial changes in their masculine views, particularly with the regard to trust and openness with others. This experience has implications for men realising their ability to engage others and for community well‐being.

## 1 INTRODUCTION

**What is known about this topic?**

- Adult male relationships promote well‐being and a sense of belonging for men.
- Many men are uncertain about how to connect with other men.
- Adult male connection and sense of belonging are being facilitated by voluntary mentors.

**What this paper adds?**

- Voluntary male mentoring facilitates personal connection with other men.
- Unexpected and emergent emotions were experienced within voluntary mentoring.
- Male mentors valued the social connectivity that emerged from their mentoring relationships.

Men in Australia value connecting with men with whom they can communicate openly and safely (Huang et al., 2019). Many organisations, institutions and clubs offer men an avenue for engagement through undertaking like‐minded activities, thereby meeting the human need for social connection (Heinsch et al., 2022). In recognition of the positive impact social connection has on men's well‐being, there are not‐for‐profit (NFP) organisations linking men to an array of programs (AMHF, 2022). These include the national (Australian) Men's Shed Association, in which men work ‘shoulder to shoulder’, then meet around the lunch table in an inclusive and supportive environment (Anstiss et al., 2018; Mackenzie et al., 2017; Waling & Fildes, 2017; Wilson & Cordier, 2013). Men are volunteering to organise and actively engage with each other, such as going for a group walk with the motto of ‘no man walks alone’ (TheManWalk, 2022), or attending a regular monthly dinner commitment to meet with others within an accepting framework (Men'sTable, 2022), or participating in a one‐to‐one mentoring dyad for a minimum of 6 months (MentoringMen, 2022; Mentors4Men, 2022). These programs aim to offer men autonomy, new social connections and a sense of belonging, which are often favoured by men in forming new relationships (Cattan et al., 2005; Holt‐Lunstad, 2021; Noone & Yang, 2021).

Nonetheless, over decades, ordinary people, often with good mental health and capable social skills, can identify as lonely even whilst being among others (Fidler, 1976; Franklin et al., 2018; Perlman & Peplau, 1981). Many men experience loneliness which may influence suicidal ideation, which often emerges from changes across the life‐course, particularly when employment changes, sporting participation ceases or relationships break down (Guntuku et al., 2021; Lim & Australian Psychological Society, 2018; Patulny & Wong, 2013). Although all genders experience loneliness, many men are unsure of how to address the condition, despite being able to recognise its presence in both their own and other men's lives (Arbes et al., 2014; Feo, 2013).

Gender roles, even in close circles or inclusive social contexts, may obscure opportunities for men to form a relationship of depth (Harding & Fox, 2015; McKenzie et al., 2018). Further, potential help‐seeking that may improve a man's mental health may inadvertently be perceived as policed or contested (McKenzie et al., 2022). Permission for men to express emotionality, often interpreted by men as a feminine trait, may conceal distress and vulnerability (Connell & Messerschmidt, 2005). Connell's earlier writings distinguished between the normative or ideal behaviour and the commonplace or typical behaviour, thereby highlighting the deliberation of who decides what should be seen as ‘appropriate’ (Connell, 1997). However, masculine traits or norms of behaviour are fluid as a society and individuals change narratives regarding socially appropriate behaviour (Adegbosin et al., 2019; O'Brien et al., 2005). The stoic man who (mentally) suffers in silence rather than inconvenience others with his perceived weakness may be less acceptable when media and politics present help‐seeking messages as mainstream (Movember, 2022). Transgressing ideals of self‐reliance and men seeking the company of other men to connect at a deeper level may be seen as suspect or subordinate in some circles, such as among hi‐vis wearing labourers, whereas other men can and do embrace counter‐hegemonic masculinities from which they draw benefit (Anderson & McCormack, 2016; McKenzie et al., 2018; Trail et al., 2021). This is exemplified by a QR code sticker campaign placed in port‐a‐loo cubicles on construction worksites to encourage men ‘having a crap day’ to connect privately for mental health support services (HALT, 2022). Significantly, Arbes et al. (2014) identified voluntary peer‐led interventions as having the most traction in reaching socially disconnected men, with informal mentoring a pivotal opportunity to enhance some men's life satisfaction (Schafer & Upenieks, 2016).

Despite this traction generated by voluntary peer‐led interventions, there are limited systematically reviewed scholarly articles, peer‐reviewed books and authoritative reports on men's experience of mentoring in voluntary situations (Henderson et al., 2022). A scoping review by Henderson et al. (2022) of voluntary male mentoring revealed the primary theme of an intentional relationship based on human connection and care (Arora & Rangnekar, 2015; Butera, 2008; Emlet & Harris, 2020), drawn from experiences of altruistic support (Ayton & Joss, 2016; Brady et al., 2017; Celdrán et al., 2018; Rahja et al., 2016; Yuen, 2002; Zucchero, 2011). Volunteer relationships of depth contrasted with mentor experiences from institutions and industry in that emotional learning unexpectedly occurred, particularly in relation to mentors' social self‐confidence (Greenwood & Habibi, 2014; Sanchez & Ferrari, 2005; Santini et al., 2020; Shapira‐Lishchinsky & Levy‐Gazenfrantz, 2016; Smith & Greenwood, 2014). The implications are that capability for male connection is present in our communities, and the benefits are two‐way in a one‐to‐one relationship. Yet adult male to adult male social connection is reported by some men as feeling awkward and difficult to progress (Arbes et al., 2014). The review revealed a lack of research and no Australian studies on the lived experience of male volunteers who intentionally engage in male social connection outside of support roles and interest groups (Henderson et al., 2022).

This article reports the lived experiences of voluntary male mentors in Australia who have formed relationships with other adult men. Mentors' experiences of facilitating one‐to‐one male mentoring relationships and the emergent products were recorded. The findings are presented as a thematic synthesis using the narrative data from interviews with mentor participants. These findings provide insight into male relationship formation over time. The discussion in this article explicates the men's narratives using a phenomenological approach and drawing on hermeneutic methodology. This study aimed to generate an understanding of men's ability to facilitate relationships with other men and the emergent products of these interactions based on the lived experience of voluntary male mentors.

## 2 METHOD

### 2.1 A hermeneutic phenomenological methodology

Phenomenology is a philosophy and qualitative methodology that focuses on an individual's lived experiences within the world (Heidegger, 1962; Neubauer et al., 2019). Through researching lived experiences toward phenomena, a fuller grasp of what it means to be in the world may be described, drawing from lived experiential narratives of everyday existence, such as one‐to‐one meetings between men (van Manen, 2016). The use of phenomenology was important to this study because it provided a lens for a subjective understanding of men's relational experiences, whilst inherently adopting an exploratory approach (Moustakas, 1994). Men have previously reported hesitancy in forming relationships with other men; therefore a phenomenological approach uncovers the mentor's experience facilitating relationships of depth in the ‘life‐world’ as it is lived (Arbes et al., 2014; van Manen, 2016). Hermeneutic phenomenology, described as a combination of textual interpretation and the study of the meaning of phenomena, informed the interpretation of the narrative data and the thematic identification (Castleberry & Nolen, 2018; Laverty, 2003; van Manen, 2016). This framework was significant in terms of discerning the lived experiences of voluntary male mentors with regard to making sense of their mentorship experiences.

### 2.2 Sampling and recruitment

Ethics approval was granted by the University's Human Research Ethics Committee (Approval Number: 2020/122). Recruitment was through purposeful sampling from Australian mentoring organisations via their executive to request their members' involvement in the study. Twelve men were selected by the executives based on having mentored another adult man in an unpaid voluntary capacity for at least 6 months (Nowell et al., 2017). The relationship typically had boundaries such as no material exchange and an absence of transactions, transportation and home visits. It was not extended to other people or social events. The men organised their meeting format and times, agreed on aspects such as confidentiality, and could discuss anything of interest. They met with their mentee face to face, sometimes using telephone and internet during Covid‐19 restrictions. Texting was mainly used for a brief check‐in or to encourage contact, particularly with younger mentees. Mentoring occurred in a range of indoor and outdoor settings including cafes, parks and walking trails.

The participants were advised of the research, interview format, the management of the data, and then signed and returned a form consenting to be included in the study (Minichiello et al., 2008). Convenient times were organised over an eight‐week period during which they were interviewed individually. They lived in urban, regional and rural settings across four states of Australia, presented as cisgender, and ranged in age from 48 to 79 years. Some men were first‐time mentors with 6 months experience, whilst others had mentored in a variety of situations for many decades. All had engaged with some form of mentor‐facilitated development and most men could identify previous or current mentors in their own lives.

### 2.3 Data collection

The mentor's lived experience of mentorship, from their own perspective, was the stated focus of the interview, which was conducted online via the Zoom video platform. The interview was conversational and comprised open‐ended questions with various prompts to elicit more expansive information (van Manen, 2016). During the interview, often the interviewer repeated the participant's language as a method to draw out more of the participant's experience on any given area, and provided participants with the opportunity to refine or clarify their responses (Minichiello et al., 2008). In addition to transcribing the verbal interaction (Groenewald, 2004), expressiveness, body language, reflective pauses and utterances were treated as relevant data and were noted on the transcript (Goffman, 2002). Interviews ranged in length from 42 to 65 min, including a brief welcome, unstructured time for participants to reiterate or reflect, and concluding goodbyes.

The researcher, as a previous workshop facilitator in mentor development, was an insider in the research process, which from a hermeneutic phenomenological perspective, means the values of the investigator inevitably influenced this inquiry (Denzin & Lincoln, 1998). Prepared semi‐structured questions assisted the researcher to remain reflective, insightful, sensitive to language and constantly open to the participant's experience during the interview (Braun & Clarke, 2019; van Manen, 2016). Sense‐making and meaning involved in the interpretation of information by the interviewer were assisted by the use of reflective journals, and the social and cultural contextual oversight contributed by the academic team (Denzin & Lincoln, 1998).

### 2.4 Thematic analysis

Thematic analysis combined a researching process and a writing process with constant referencing to the research objectives (van Manen, 2016). Through a process of immersion, the participants' narratives were examined. Structured steps of analysis began with familiarisation of the data via watching, listening and transcribing the video‐recorded interviews. Topic areas significant to the participants, as demonstrated through their voice and expressions, were manually noted on hardcopy which formed the first group of categories. The transcribed data were moved to NVivo software (QSR_International, 2021) to assist in data management, and were reviewed line by line with additional categories emerging. As a qualitative interview, repeated information was summarised in the codification process, thereby refining and reforming some categories (Clarke et al., 2015; Minichiello et al., 2008). The data in this phenomenological study were then returned through a writing, reading and editing process that moved through multiple iterations (Neubauer et al., 2019). The second, third and fourth authors examined the data independently and then met to discuss. Codified data were cross‐referenced with regard to the specific interview question to verify that the categorised data reflected the participants' meanings.

Codes were connected as a process of discovering themes and patterns in the data (Groenewald, 2004). Categories were merged, and codes were subsumed to present the mentors' experience. The lead author then synthesised preliminary themes, explicating contrasts and resemblances across the data. Male gender norms were recognised as occurring in the descriptions of the voluntary male interactions provided by the participants, with representative quotes exemplifying this gendered experience in the study (Butera, 2008). Themes were further refined by all the authors, and data were then presented using derivates of the mentor's language and expressions (Nowell et al., 2017; Thomas & Harden, 2008).

## 3 FINDINGS

Three themes encompassed the expressive and narrative data from the lived experience of voluntary male mentors in Australia. These themes are presented as ‘Connecting through Listening’ and ‘Being Real’, which facilitate satisfying male relationships, and ‘Empathic Awareness’, which encapsulates the emergent products of this relationship for the mentor.

### Theme 1: Connecting through listening

Connecting through listening was a palpable and essential undertaking within the voluntary mentorship experience. Mentor A was forthright in stating, “if there is a focus… you've got to connect”; it was the critical tenet of mentorship: “connection…it's a core”. Mentor H, speaking slowly and precisely as he found his words, said, “you make an effort, you're willing. And this is another important part about mentoring; a willingness to connect with a stranger.” The effort and commitment of voluntary mentoring was understood by many of the mentors as an essential component of connection. Mentor H spoke passionately about connecting but acknowledged masculine reticence:

“To me mentoring is about connecting with another man, connecting with a person as an equal. In life we are social creatures, and life is all about connecting with other people… and just that, there's the purpose with mentoring… it's easier, particularly for a man not to connect … easier to maintain your barrier”.

Mentor W stated that connecting with others “is a gift a lot of people don't have”, and Mentor I recognised the necessity to “not give up on them and say oh this is all too hard. Be less transactional and get involved in their life.” Mentor R, rubbing his brow with an extended pause, described his effort as follows:

“I think it is just you want to do it. You've got to be a person that wants to be a mentor, or wants to help. And if you have that want, then, I suppose we've all got our own methods. But it's just a want.”

Each mentor discussed listening at length throughout their interview. Voluntary mentors listen, and listen some more, as expressed by Mentor C, “You are here to listen…I'm constantly reminding myself of that”. Mentors applied themselves to the process of active listening, demonstrating a concentrated effort as declared by Mentor W, “I'm still, I'm still, I'm still listening”, then slowly shaking his head with an extended silence; “you just go, all right—you're there. They're ready to unload.” Listening was an activity shared, sometimes in silence, with another man. Mentor G put it succinctly, “just there to listen, and you know, the man was really happy just to talk”. Mentor T, like most participants, reflected on his own skill improvement, “just be there, in an encouraging way…and I've learned to listen more, and not speak.” Listening at times required effort and focus which went beyond words to include the whole person, as Mentor R voiced, “So I listen more intently to people. What they are trying to say—or if I can see a feeling in a person – if that makes sense.” Mentors listen for facts and feelings.

Some mentors recognised that a leading question could be thinly veiled advice in a different form. Mentor C, grinning and stooping his head, offered, “go and do X, Y and Z. I might be thinking those things, but I don't say it”. Mentors reported that this breaks the connection. Advice was identified as not listening. Mentor W stated, “I'm not there to judge. I'm only there just to listen”, and Mentor F said, “One of these makes you, at least, less effective. You see, if you try and fix the other person, solve the problems”. Mentor B referring to a mentor development workshop he attended “that poem, *Please Listen*… When I ask you to listen and you proceed to give me advice, you haven't heard, what I really asked you to do and you are trampling on my feelings… that sort of thing” (Houghton, 1979).

### Theme 2: Being real

Being Real is a theme that captures the essential elements of genuineness and equality between men, shared in each other's company, in the moment. The mentors expressed various feelings which may be described as real, such as an “authentic relationship” (Mentor K), “being genuine” (Mentor A), “experiencing perspective” (Mentor W), “sharing deeply” (Mentor M), in a “confidential and safe environment” (Mentor K), and “really talking and building caring trust” (Mentor M). Mentor T described his time with his mentee as follows:

“This conversation is not time‐pressured. I can allow you to talk as much as you want, and I can wait and think about my response as long as I want, because we're in each other's company, as two human beings.”

In poetic terms and referring to his acting background, Mentor C expressed, “Now the lines are written, but actually the intent is much more real if you're responding in the moment to what is being said or done to you.” Mentors were not directing another person; as stated by Mentor I, “It's letting people find their own answers”. Many of the mentors recognised the uniqueness of a one‐to‐one voluntary relationship with both parties “feeling valued…feeling appreciated” (Mentor C) and “equal… and not telling him what he should do” (Mentor H). The relationship was not contested, and its benefits were shared. Mentor G expressed awareness of setting aside his own value judgements when volunteering in a mentorship setting, when he said:

“So, the rush to judgment, you know, the rush to verdicts, you know, that kind of tendency with all of us, I guess, but with myself, of course, so to hold back on that, and to maintain that focus of, look, ‘I'm not here to judge.’ I'm not here to condone behaviour or otherwise, so I'm here just to listen and to provide an outlet for the other guy to talk. Yeah.”

Mentors made a significant contribution by being available for others to confide their pain. Mentor I stated: “Helping them to find different pathways. I'll be helping people just see there are other ways, other than suicide in some cases.” Mentor T was direct about his experience of male distress when he said, “I looked for being able to somehow make a contribution to the improvement of men's lives, but also from an attitude that it would be in a form that it might assist with suicide prevention.” Mentor H, very seriously voiced:

“I believe, in a small way, we're tackling a major problem of men's suicide…and if men feel better about themselves, I believe there's a ripple effect, and that goes out into the community, and it helps people make changes…it generates positivity in a community which is lacking in some people's lives.”

Mentor A, somewhat hesitantly but with passion, stated, “the issue can be solved by more love, by more connection, by reducing loneliness. It's what mentoring does.” Mentor W stated, “I'm a much calmer person. I don't take on other people's issues,” which was similar to Mentor R when he said, “So I've become more relaxed about issues, so what, like grass grows, I don't worry about the less important things”. Mentors developed their approaches to stay connected during challenging conversations and were developed by the experience, as stated by Mentor M, “that teachers me as a mentor…to really try and understand a wide variety of other people”.

### Theme 3: Empathic awareness

Empathy and forming new perspectives were repeatedly referred to by the mentors as changing them. Mentor K said with enthusiasm, “They have value, they have intellect, they have whatever, their experiences are so different to mine… I'll try to get into their zone.”

Many of the mentors experienced a changed awareness, described as “opened up”, “energised”, “calmer” and having “developed confidence”. Mentors A, C and W identified personal development by being able to speak to just about anybody with empathy and more understanding. Mentor A stated, “I think it's made me a better person, a more empathetic person.” Mentor W laconically attributed significant personal changes resulting from his mentoring:

“Those life skills in personal development. Being able to talk to just about anybody. Not feeling embarrassed about what I'm talking about. Seeing other people's opinions, views on life, politics, that sort of thing. So, it's just such a huge subject, but yeah, I just see it in other people, and how they respond to me now.”

Each mentor expressed satisfaction, contributing or doing something of meaning in their life as an important aspect of the relationship. Mentor M drawing on his decades of experience in mentorship, and reflecting on the other person, the mentee, stated:

“And you don't need a degree to be a mentor, you need a life experience, communication, empathy and a bit of patience… being able to really sit down and go somebody who doesn't think the way I think at all. And you sit down and go, you're doing this for a reason? I need to work out why and that's helped me in so many different areas.”

Mentorship had surprises for many of the participants. Literately voiced independently by Mentors A, C, D, H, I, K: “How can I help more?” was an unexpected internal question that arose in these men. They either stated or inferred that voluntary mentoring is “good for my mental health.” Meeting and connecting with “strangers” (Mentor C) or people that were “not on my radar” (Mentor I) had introduced circumstances that were stated as new to many of them with “caring and trust” (Mentors A, C, D, H) collectively used to express unanticipated feelings in their mentoring relationships. Most participants were enthusiastic about being in a mentoring relationship, as captured in the statement by Mentor R, “If I took it away. If I took it out of my life, I would be looking to replace it. I'd have a hole in my life.”

## 4 DISCUSSION

Voluntary male mentoring is a rich and unique human interaction requiring intimate expression and one‐to‐one connection between a mentor and a mentee. The three themes presented from mentors' lived experiences share similarities with person‐centred humanistic psychology. Active listening, unconditional positive regard and trust are framed within a widely adopted therapeutic process that has proven beneficial to many people (Rogers & Farson, 1957). However, significantly for community well‐being, voluntary male mentors are unqualified as health professionals yet able to facilitate relationships beneficial to men (Eversole, 2012).

The first theme, Connecting through Listening, presents the facilitation of voluntary male mentorship as an active process understandable by men in the wider community. Listening is a skill that improves with practice and may be extended with exercise and training (Rogers & Farson, 1957; Zucchero, 2010). Men in the community are able to both speak and listen (Paul et al., 2022). Advice is mostly with‐held and treated by mentors as an internal signal to re‐focus their present intention, without judgement, to their mentee. This contrasts significantly with the workplace and institutional mentoring where advice giving is a key tenet. (Appelbaum et al., 1994; Chao et al., 1992). The mentors gave connection in the relationship primacy.

Mentors pointed to the requirement of wanting to connect with another person. They appreciated the flexibility of being an unpaid volunteer and that mentoring engaged them in their community. They understood mentees were probably distressed and the relationship would require behaviours seldom associated with man to man interactions (Anderson, 2009). Following trust‐building and associated deep listening, the mentee was likely to disclose issues affecting them (Granato et al., 2015). Some mentors acknowledged masculine difficulty in acting openly, yet recognised this vulnerable expression as being formative of a connection described as authentic. The mentors' voluntary contribution encouraged a previously unexercised intimate form of personal acknowledgement between the men (Holmes, 2015). Appreciating and valuing others by connecting through listening, was perceived to generate positivity in their community, and that felt good for the mentors.

Being Real, the second theme, carries the concept of being present in the lived world (van Manen, 2016). In this study, mentors were present in a dyadic intentional relationship. It is the mentee's alterity, or state of being other, which completes the mentorship (Merleau‐Ponty & Smith, 1962). The mentors did not reduce the other man to a set of conditions or define them by an issue. They had to set their tendency to rush to judgement aside, and remain connected with the mentee. They were engaged in a first‐person relationship that was not time‐pressured or outcome‐focused. Mentors were sharing in an equal relationship that felt genuine.

The mentors acknowledged the emotional distress of mentees as often related to loneliness. It may have been proceeded by the loss or lack of inclusion in a relationship and sometimes presented with suicidal ideation. The experience of these interactions challenged the mentors to remain present and listen to the other man. These significant interactions instigated personal deliberation for mentors that questioned the relative importance of issues. The mentors were required to try and understand matters that previously in their life they may have thought less relevant. They were grateful for exposure to this form of effort and its developmental attributes.

Care, trust and empathy emerged from the mentoring activity. On reflection, the mentors recognised personal development in open honesty, personal empathy development and more intent in their lives. They were changed by the experience, which is thematically described as Empathic Awareness. Some participants listened for feelings and felt they addressed loneliness in others and themselves.

Volunteering has been positively associated with a sense of well‐being and belonging (Keyes & Waterman, 2003; Yuen et al., 2008). The voluntary mentors who participated in this study expressed a range of emotions, sometimes unexpected, that may be described as joyful and having meaning, reflecting a flexible masculine identity (Waling, 2019). Mentors experienced growth in their mentorship, particularly in feelings of empathy for themselves and others. Voluntary mentorship as an indirect form of interpersonal understanding gained from a face‐to‐face experience was an authentic encounter for the men (Zahavi, 2018). They reported improved interpersonal skills by communicating more openly and with a broader group of people.

The participants in this study perceived they had the capacity to volunteer and contribute to men's well‐being. Their lived experience of voluntary mentorship occurred in a unique setting therefore the findings cannot be generalised to other individuals. However, the relevance for broader insights in facilitating male relationships, focusing on connection and listening, rather than advice or judgement, holds relevance for men. Communities may be informed by the experience of voluntary male mentors in their one‐to‐one masculine interactions.

## 5 CONCLUSION

Voluntary male mentors keep connecting throughout their interaction and are active listeners of other men's feelings. Their mentorship activity provided the participants with a sense of contribution, personal growth and an emerging empathy. These relationships offer new lines of inquiry to explicate changing masculine gender norms as experienced by men in their communities. Participants experienced personal growth by developing relationships that proved satisfying to them. Furthermore, the voluntary mentors identified masculine empathy was counter to many of their previous experiences with men and warrants further inquiry. This study generates an understanding that voluntary peer‐led interventions assist men to form satisfying relationships over their life course.

## FUNDING INFORMATION

No financial or material support has been provided in the production of this article.

## CONFLICT OF INTEREST

The authors report no conflicts of interest.

## Data Availability

Research data are not shared.
